# I do not want to suppress the natural process of inflammation: new insights on factors associated with non-adherence in rheumatoid arthritis

**DOI:** 10.1186/s13075-018-1732-7

**Published:** 2018-10-19

**Authors:** Valentin Ritschl, Angelika Lackner, Carina Boström, Erika Mosor, Michaela Lehner, Maisa Omara, Romualdo Ramos, Paul Studenic, Josef Sebastian Smolen, Tanja Alexandra Stamm

**Affiliations:** 1 Section for Outcomes Research, Centre for Medical Statistics, Informatics, and Intelligent Systems, Medical University of Vienna, Spitalgasse 23, 1090 Vienna, Austria; 2 Division of Rheumatology, Department of Medicine 3, Medical University of Vienna, Vienna, Austria; 3 Department of Rheumatology, Medical University of Graz, Styria, Austria; 4 Division of Physiotherapy, Department of Neurobiology, Karolinska Institute, Care Sciences and Society (NVS), Huddinge, Sweden; 5 Karolinska University Hospital, Stockholm, Sweden; 6 Department of Internal Medicine, Centre for Rheumatic Diseases, Hietzing Hospital, Vienna, Austria; 7 Division of Occupational Therapy, University of Applied Sciences FH Campus Wien, Vienna, Austria

**Keywords:** Qualitative research, Deep understanding of patients’ perspectives, Rehabilitation

## Abstract

**Background:**

It is estimated that 50–70% of patients with rheumatoid arthritis (RA) are non-adherent to their recommended treatment. Non-adherent patients have a higher risk of not reaching an optimal clinical outcome. We explored factors associated with nonadherence from the patient’s perspective.

**Methods:**

Four hundred and fifty-nine RA patients (346 (75.4%) females; mean age 63.0 ± 14.8 years) who failed to attend follow-up visits in two rheumatology centres were eligible to participate in a qualitative interview study. We used this strategy to identify patients who were potentially non-adherent to medicines and/or non-pharmacological interventions. By means of meaning condensation analysis, we identified new and some already well known insights to factors associated with non-adherence. We used the capability, opportunity, and motivation model of behaviour (COM-B) model as a frame of reference to classify the factors.

**Results:**

Forty-three of 131 patients (32.8%) who agreed to participate in the qualitative interviews were found to be non-adherent. New insights on factors associated with non-adherence included strong opinions of patients, such as pain being considered as an indicator of hard work and something to be proud of, or inflammation being a natural process that should not be suppressed; feeling not to be in expert’s hands when being treated by a physician/health professional; the experience of excessive self-control over the treatment; and rheumatologists addressing only drugs and omitting non-pharmacological aspects. The COM-B model comprehensively covered the range of our findings.

**Conclusions:**

The new insights on factors associated with non-adherence allow a better understanding of this phenomenon and can substantially enhance patient care by helping to develop targeted interventions.

**Electronic supplementary material:**

The online version of this article (10.1186/s13075-018-1732-7) contains supplementary material, which is available to authorized users.

## Background

Rheumatoid arthritis (RA) is a chronic inflammatory disease characterized by destructive synovitis [1]. RA has an important impact on daily functioning including work capacity, social participation, and quality of life [2, 3]. The main target of treatment is to control disease activity [4], to reduce symptoms, to decrease the daily impact of the patients’ condition, and to increase the feeling of a return to normality [5].

Disease-modifying anti-rheumatic drugs (DMARDs) reduce disease activity and radiological progression and improve long-term functional outcome in patients with RA [6]. However, it is estimated that 50–70% of patients with RA are non-adherent. These patients do not follow the recommended treatment/prescriptions [7–10]. Many of these will not achieve an optimal clinical outcome, since not taking medication as recommended is associated with more frequent disease flares and increased disability [11, 12]. Therefore, improving adherence enhances the efficacy of medical treatments and reduces hospitalisation and the subsequent healthcare costs associated with RA [8, 9, 13–16].

Non-adherence is a phenomenon which exists independent of age, gender, socio-economic status, health condition, setting, and/or prognosis [7, 17]. Furthermore, non-adherence can occur in the initial treatment phase (late or non-initiation of the prescribed treatment) and/or in the later treatment phases (sub-optimal implementation of the dosing regimen or early discontinuation of the treatment) [8, 18]. Non-pharmacological methods are associated with even lower adherence rates compared with medication because they often include life-style modifications and thus require changes in behaviour and habits of daily routine which are difficult to achieve [19].

The complexity of non-adherence is addressed by the psychological theory of planned behaviour [20], which posits that attitudes, subjective norms (i.e. expectations of others), and behavioural control are determinants of our intentions and subsequent actions. More specifically, scholars have recently suggested frameworks such as the capability, opportunity, and motivation model of behaviour (COM-B) [21] to describe patient (non-)adherence. The COM-B is a comprehensive model designed to understand human behaviour and includes capability (the physical and psychological capacity to be adherent, such as memory or comprehension of disease and treatment), opportunity (the physical and social factors to make adherent behaviour possible or prompt it such as access to healthcare facilities and regime complexity), and motivation (brain processes that energise and direct behaviour, such as perception of illness and beliefs about treatment) [22]. The model acknowledges that behaviour is part of an interacting, dynamic system involving these three components to determine a person’s behaviour and, in this particular case, medical adherence. This is in accordance with the International Classification of Functioning, Disability and Health (ICF) put forth by the World Health Organization (WHO) [23]. While the contextual factors of the ICF are designed to explain functioning in a health-related context, the COM-B focuses on behaviours in any context that influences a person’s engagement in activities and participation. The COM-B provides a more in-depth understanding than other widely used models such as the necessity-concerns framework [24, 25] and binary models of intentional and unintentional non-adherence by including not only patient’s beliefs, but also physical, cognitive, and environmental determinants of behaviour [26]. A further advantage of the COM-B model when compared with other approaches is its applicability in interventions, such as evidence-based behavioural change techniques [27, 28]. The model has garnered support in recent literature [29, 30].

According to the WHO, the perspective of patients, including motivation, values, beliefs, and needs, are essential factors that influence non-adherence [9, 31]. However, there is still a lack of deep qualitative data regarding the range and variability of motivations of patients not to adhere. Furthermore, some studies did not differentiate between early and late phases of treatment [32], while others did not explore reasons why patients with RA did not show up for regular follow-up visits [30, 33, 34]. In the area of non-pharmacological methods, the knowledge on the perspectives of patients is even more limited; only case reports [35–46] exist, and no studies have systematically investigated the perspective of these patients in greater depth. None of the cited studies cover the multi-faceted nature of non-adherence as described in the model above [9]. The rigorous use of qualitative research methods is an ideal means to investigate the perspective of patients in a scientific, systematic way. Qualitative research methods investigate in depth the perspectives, motivations, values, beliefs, and needs of patients [47]. The findings of qualitative studies can inform subsequent quantitative models at a later stage.

In the present study, we therefore aimed to explore factors associated with non-adherence regarding medication and non-pharmacological methods from the perspective of patients with RA covering the earlier and later phases of treatment. Furthermore, we aimed to systematically report for the first time self-reported reasons for non-adherence to follow-up visits in a large sample.

## Methods

### Study design

Our study was performed in two parts. First, we identified potentially non-adherent patients and extracted their clinical data retrospectively using a database query at two rheumatology centres in Austria. Second, we invited these patients to participate in a qualitative interview. Based on the qualitative interviews, patients were assigned to having been non-adherent when they reported that they had stopped seeing a rheumatologist and/or were taking less than approximately 80% of the medication (steroids and DMARDs) prescribed [9]. From the perspectives of the non-adherent patients, we identified factors associated with non-adherence. We compared these factors with the literature to triangulate our findings, and we used the COM-B model [21] as a frame of reference to classify the factors we identified. The ethical committees of each institution approved the study (EK 1082/2015 (Vienna) and 27–324 ex 14/15 (Graz)). Reporting of the qualitative results was done according to the COREQ guidelines (Additional file 1: Supplement S1).

### Identification of patients and extraction of clinical data

To identify potentially non-adherent patients, retrospective, observational data from patients with RA (EULAR/ACR criteria) [48] were selected from the databases of two rheumatology centres in Austria (Graz and Vienna); inclusion criteria were: 1) non-attenders to follow-up visits at the rheumatology centre over a time period of at least 9 months; 2) had a minimum of four visits; and 3) at least one prescribed DMARD. The identification of non-adherent patients was an essential aspect of our study. We therefore selected potentially non-adherent patients based on the fact that they did not attend regular follow-ups. This was considered a new and different identification strategy in contrast to asking patients consecutively in the outpatient clinic whether they were adherent or not because we expected a large number of socially desirable answers. To further reduce such reporting bias during the qualitative interviews, all interviews were performed by health professionals (VR, male, MMSc, health scientist with a background in occupational health and therapy, as well as assistive technologies; and AL, female, PhD, MSc, nursing scientist) who were not involved in the patient care or otherwise related to the patients. The following data were extracted from the last clinical visit of each patient: swollen joint counts (SJC32) and tender joint counts (TJC32) using 32 joint counts, erythrocyte sedimentation rate (ESR; mm/h), C-reactive protein (CRP; mg/l), anti-citrullinated protein antibodies (ACPA; U/ml), rheumatoid factor (RF; U/ml) [49], score of the Health Assessment Questionnaire (HAQ) [50, 51], patient global assessments (PGA) and evaluator global assessments (EGA), and pain using 10-cm visual analogue scale (VAS). Clinical and Simplified Disease Activity Indices (CDAI and SDAI) [52–54] were also calculated.

After comparing our data with the clinical case records and the death data registry to eliminate potential other reasons for non-adherence, such as significant other disease or death, we contacted all identified, remaining patients via telephone, informed them about the purpose and procedures of the study, documented self-reported reasons for non-adherence to the follow-up visits, and invited them to participate in a qualitative semi-structured interview (conducted from April 2015 to February 2016). If a patient gave oral and written consent to participate in this study an appointment for a one-time, one-on-one interview was made according to the preferences of the patient either in the course of a (second) scheduled telephone call or a face-to-face interview at the clinic. In case patients could not be reached, two researchers (VR and AL) tried to contact these patients three times at different time points in a day. If this procedure was not successful, they were considered not reachable.

### Data collection

An interview guide was developed for the semi-structured individual interviews using the capability, opportunity, and motivation of the COM-B model [21] as a frame of reference. The interview guide was reviewed and adapted by the patient research partner (ML). Questions focused on the current status of rheumatology care of each patient, potential reasons for non-attendance to the follow-up visits and non-adherence to prescribed DMARDs and/or non-pharmacological methods. Examples of interview questions were as follows: “Please describe your experience from your last visit at the rheumatology centre?” (COM-B domain: motivation); “Which reasons prevented you from regular follow-up visits at the rheumatology centre?” (COM-B domain: motivation, opportunity); “Which medications were prescribed at your last visit in our centre and which of these do you still take?” (COM-B domain: opportunity); “Have you experienced any side effects due to your medication?” (COM-B domain: motivation, capability/body structures and functions); “What was your experience with non-pharmacological prescriptions/instructions?” (COM-B domain: motivation, capability/body structures and functions); and “Did you implement any of these recommendations in your daily life?” (COM-B domain: motivation, opportunity). The whole interview guideline is depicted in Additional file 1: Supplement S2.

### Qualitative data analysis

Qualitative data were analysed using a meaning condensation analysis [47]. First, the audiotaped interviews were transcribed. If participants provided information during the first telephone contact, field notes were taken. The transcripts and potential field notes taken during the interviews were read through to gain an overview of the collected data. Second, the data was divided into meaning units (defined as specific units of text, a few words, or a few sentences with a common meaning). Meaning units represented the range of patient experiences. In a third final step, the concepts contained in the meaning units were identified. An example to illustrate the procedure of the qualitative analysis is shown in Additional file 1: Supplement S3. The concepts depict the factors associated with non-adherence identified in our study.

Subsequently, we assigned so-called “time-tags”, when patients explicitly mentioned a specific time in their treatment course of the disease when an event of interest occurred, and “pharmacological versus non-pharmacological tags”, when patients specifically related aspects to one type of intervention, e.g. adherence may be different in taking medication compared with performing and motivating oneself to perform exercises. Thereafter, we linked the factors extracted from the qualitative analysis to the domains of the COM-B model [21], namely capability, opportunity, and motivation.

Based on the qualitative interviews, patients were assigned to having been either adherent (having self-reported regular rheumatology visits in another centre or with a rheumatologist and were taking approximately 80% of the medication related to glucocorticoids and DMARDs as prescribed) or non-adherent (having stopped seeing a rheumatologist and/or taking less than approximately 80% of the medications related to steroids and DMARDs as prescribed) [9].

To ensure accuracy and rigour of the qualitative analysis, all interviews were performed according to a pre-determined interview guide; 25% of the results were reviewed by a second researcher (either TAS, EM, or MO) who have extensive experience in the field of qualitative research prior to the present project [47, 55, 56]. In addition, a patient research partner (ML) reviewed the results. In case of disagreement, the results were discussed (by VR, TAS, EM, MO, and ML) until consensus was achieved to obtain a common understanding about the meaning of the data and depth of the concepts.

### Descriptive statistics

To summarize categorical variables, we used absolute frequencies and percentages. Discrete or continuous variables were described in terms of mean and standard deviation. The analysis was conducted using SPSS 24 (IBM) [57].

## Results

### Patient characteristics and reasons for non-adherence to regular follow-up visits

**Fig. 1:**
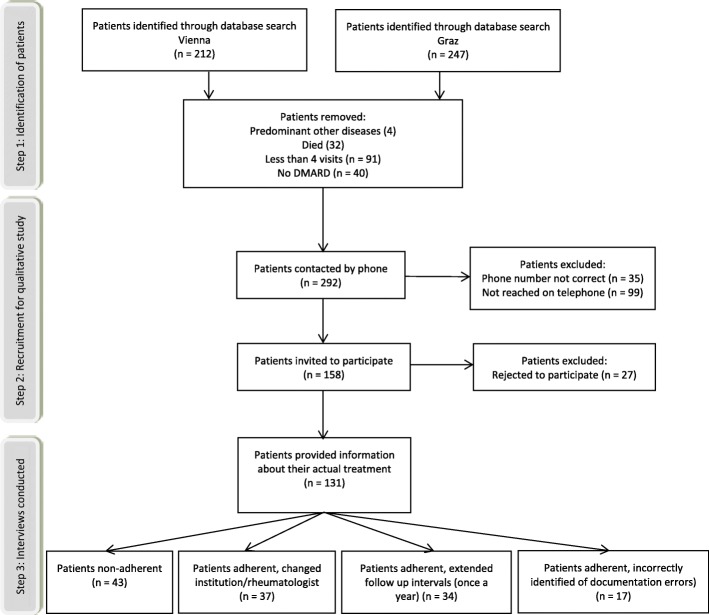
Patient flow chart, showing the results of the database query and the procedure for patient selection for the study. DMARD disease-modifying anti-rheumatic drug

Of the 459 identified patients, 32 (7%) had died; 4 (0.9%) had a predominant other disease, including malignancies (1; 0.2%), dementia (2; 0.4%), or were in palliative care (1; 0.2%); 134 patients (29.2%) could not be reached; 27 patients (5.9%) rejected participation in the qualitative interview; and 131 (28.5%) did not fulfil the inclusion criteria due to documentation errors despite the fact that they had been identified in the database query (Fig. 1). A total of 131 (28.5%) patients agreed to participate in the qualitative study. Interview duration ranged from a few sentences up to a maximum of 32 min.

Based on the qualitative interviews, 43 (32.8%) of the 131 patients were classified as non-adherent and 88 (67.2%) were found to be adherent. Of the 88 adherent patients, 37 (42.0%) changed the centre or rheumatologist, 34 (38.6%) had extended (annual) intervals at the clinic, and 17 (19.3%) were incorrectly identified as non-adherent because of documentation errors. The characteristics of the adherent and non-adherent patients who participated in the qualitative interviews are presented in Table 1. Baseline characteristics of patients who agreed to participate in the qualitative interviews and the non-participating individuals are depicted in Additional file 1: Supplement S4.

**Table 1:** Baseline characteristics of the adherent and non-adherent subgroups

	Non-adherent^a^	Adherent^b^	*p* value
Number of patients^c^, *n* (%)	43 (32.8%)	88 (67.2%)	–
Female, *n* (%)	36 (83.7%)	73 (83.0%)	0.912
Age (years), mean (±SD)	58.3 (±13.1)	64.1 (±13.3)	**0.014**
Disease duration ( years)^d^, mean (±SD)	10.9 (±7.6)	12.4 (±9.3)	0.792
Treatment duration (years)^e^, mean (±SD)	9.5 (±7.3)	8.8 (±7.2)	0.549
HAQ, mean (±SD)	0.9 (±0.8)	0.7 (±0.7)	0.233
SDAI, mean (±SD)	10.0 (±8.7)	6.9 (±6.4)	0.078
CDAI, mean (±SD)	9.2 (±8.2)	7.1 (±7.6)	0.121
PGA VAS^f^, mean (±SD)	29.8 (±24.7)	27.8 (±26.7)	0.453
EGA VAS^g^, mean (±SD)	14.7 (±16.2)	12.2 (±15.5)	0.325
Pain VAS^h^, mean (±SD)	30.4 (±25.3)	28.8 (±28.2)	0.554
SJC32, mean (±SD)	2.8 (±4.3)	1.4 (±2.3)	0.128
TJC32, mean (±SD)	5.3 (±6.5)	2.6 (±5.0)	**0.008**
RF positive, *n* (%)	19 (44.2%)	46 (52.3%)	0.437

Data were extracted from the last clinical visit of each patient

Metric variables are shown in terms of mean and standard deviation. For nominal variables, absolute and relative frequencies were calculated

The *p*-value was calculated using Chi-Square test for nominal variables, and the Mann-Whitney *U* Test for ordinal and metric variables; significant results are highlighted in bold

*CDAI* Clinical Disease Activity Index, *EGA* evaluator global assessment, *HAQ* Health Assessment Questionnaire, *PGA* patient global assessment, *RF* rheumatoid factor, *SDAI* Simplified Disease Activity Index, *SJC32* swollen joint count using a 32-joint count, *TJC32* tender joint count using a 32-joint count, *VAS* visual analogue scale

^a^Patients were classified as non-adherent when they reported a change in intake of medication or other prescription without consulting a professional, or when they reported taking less than approximately 80% of the medication (steroids and disease-modifying anti-rheumatic drugs (DMARDs)) as prescribed, or missing appointments occasionally (out of 131 patients; patients who died or had other predominant diseases (*n* = 36) were not assigned to the subgroups adherent or non-adherent)

^b^Patients were classified as adherent when they reported following the treatment plan and visiting the outpatient clinic (or any other institute/health professional) as recommended (out of 131 patients; patients who died or had other predominant diseases (*n* = 36) were not assigned to the subgroups adherent or non-adherent)

^c^Total *n* = 131 patients; patients who died or had other predominant diseases (*n* = 36) were not assigned to the subgroups adherent or non-adherent

^d^Disease duration refers to the time duration between the first symptoms reported by the patient and the last visit at the centres

^e^Treatment duration refers to the time duration between the first and the last visit when the patients presented themselves at the centres

^f^Patient self-report measure using a 100-mm VAS [53]

^g^In addition to the PGA, EGA integrates subjective and objective measures obtained by the evaluator [53]

^h^Measured using a 100-mm VAS

### Factors associated with non-adherence known from the literature

The following concepts confirmed earlier findings from the literature [8, 30, 32–35, 37–40, 43–46, 58], with patients being non-adherent: 1) if they did not understand the purpose of the treatment, did not experience a benefit and/or experienced adverse events and/or toxicity; 2) if the proposed treatment plan was experienced as being too time consuming, including necessary waiting times, and requiring too much effort to be implemented in daily life; 3) if a lack of support of the environment occurred; and 4) if patients were not actively involved in a shared decision-making process. A non-adherent patient described that a shared decision about her medication treatment had not taken place:

*“If the doctor does not listen to me or does not take my opinion into account when deciding about the medication that I should take, then I change the amount of the medication myself and I potentially lie to him”*; participant no. 182 (female, age 34, Vienna)

Factors associated with non-adherence known from the literature together with quotes from the interviews are shown in Table 2.

**Table 2:** Factors associated with non-adherence which are known from the literature and were confirmed in our study

No.	Factors	Description	Quotation	Domains of the COM-B model
1	Lack of understanding the purpose; no benefit and/or adverse events	Patients were less likely to follow treatment instructions if they did not understand the purpose of the treatment, did not experience a benefit, and/or experienced adverse events and/or toxicity.	*If I experience that it* [the medication/intervention] *doesn’t help or if I do not understand the purpose, he* [the rheumatologist] *must accept that the instructions are not being followed* (participant no. 182, female, age 34, Vienna). *I stopped taking the medication by myself because of severe diarrhoea. I did not wait for an appointment to consult a doctor* (participant no. 29, female, age 57, Vienna). *I am getting older and older—the age is increasingly affecting my health. Sometimes I am afraid to do the exercises because everything is more or less deteriorating—the muscles and the bones* (participant no. 110, female, age 70, Vienna).	Capability; body structures and functions
2	Implementation requirements	Patients were less likely to follow treatment instructions if the proposed treatment plan was experienced as being too time consuming, including necessary waiting times, and requiring too much effort to be implemented in daily life.	*I was personally involved in building a medical centre and therefore I had no time for regular appointments. I was very glad that I did not have to spend a whole morning at the clinic, but instead was able to solve things easier and faster by consulting friends (physicians, but not rheumatologists). I thought that it was not important to see a rheumatologist any more* (participant no. 126, female, age 38, Vienna). *I still do my exercises—or correctly spoken again. I have exercises I should do every day. I don’t do the exercises at the moment. I’m very lazy. And now I thought I could start again* (participant no. 110, female, age 72, Vienna).	Motivation
3	Lack of supportive environmental factors	Patients were less likely to follow treatment instructions if lack of support of the environment occurred.	*My mother cannot speak German. She missed the last appointment. She was not able to make a new appointment and I didn’t have time to make an appointment for her. Then I totally forgot, and that’s why she didn’t come to the outpatients-clinic* (daughter of participant no. 74, female, age 55, Vienna, who translated during the interview). *You actually have an appointment but, nevertheless, you have to wait a long time. I was afraid if I said too often that I could not come* [to work]*, I might lose my job* (participant no. 143, male, age 47, Vienna). *Meeting different doctors every time is aggravating* (participant no. 29, female, age 57, Vienna).	Opportunity
4	Lack of shared decision-making	Patients were less likely to follow treatment instructions if they were not actively involved in a shared decision-making process.	*The young doctors at the outpatient clinic were very annoying. They have no empathy. Rheumatism also has a lot to do with the soul of a patient. If young doctors consider themselves more important and think to you know everything better than the patient—that won’t work at all* (participant no. 45, female, age 57, Vienna). *At my last visit to the outpatient clinic, I felt I was not being taken seriously and I had the feeling that the outpatient clinic is not patient-centred, but instead pharmaceutical company-centred* (participant no. 37, female, age 70, Graz).	Opportunity

The capability, opportunity, and motivation model of behaviour (COM-B) model [21] was used as a frame of reference

### New insights on factors associated with non-adherence

Four new concepts emerged in our study that have not been reported so far in the literature (Table 3). The first new aspect referred to a patient’s strong opinion, meaning that values or beliefs that people accepted without any doubts inhibited adherence; pain, for example, was considered to be a necessary part of life in older age which should not be reduced because it was experienced as a reference for hard (manual) work during different phases of the patient’s life. Similarly, another participant (no. 2, female, age 40, Graz) considered inflammation to be a natural process that should not be suppressed:

*“I did not want to do this* [take the prescribed medication] *anymore. I decided that I do not always want to suppress the inflammation. I want to leave the inflammation as it is, because it is a natural process. I do not want an infusion every month that, moreover, costs so much money, which in fact only supports the pharmaceutical companies. Doctors are brainwashed by the pharmaceutical companies, otherwise they would not prescribe these drugs.”*

Second, patients felt they were not the hands of experts when being treated by a physician/health professional. This was reported when the treating physicians appeared to be inexperienced which was associated with physicians being perceived either as young regarding their age (participant no. 165, male, age 70, Vienna) or if a rheumatologist asked senior consultants or colleagues for advice during the consultation with the patient. Third, patients who perceived excessive self-control over their treatment were likely to be non-adherent. Participant no. 21 (female, age 57, Vienna) said:

*“I just started to reduce the medication on my own. And no difference was noticeable. I reduced it myself for a very long time and nothing worse happened. And that's the reason why I haven't been to the outpatient clinic for so long, because I have it under control anyway.”*

Fourth, some patients did not feel properly taken care of if the rheumatologist prescribed medicines only without giving advice on daily life issues and non-pharmacological aspects of treatment. New insights on factors associated with non-adherence together with quotes from the interviews are shown in Table 3.

**Table 3:** New insights on factors associated with non-adherence

No.	Factors	Description	Quotation	Domains of the COM-B model
1	Patient’s strong opinion, similar to a dogma	“Patient’s dogma”, meaning that strong opinions, values, or beliefs that people accept without any doubts facilitated non-adherence.	*I am 77 years old now, always worked hard and long hours. I raised 6 children and I was never unemployed. It is no wonder that I am in pain. It indicates that I have been working hard all my life* (participant no. 150, female, age 76, Vienna). *I don’t like drugs. Drugs made me sick. I never really recovered from that sickness drugs made me. I stopped taking medication. I have now bought a magnetic field mat, changed my diet and now I have no pain anymore* (participant no. 48, female, age 56, Graz).	Motivation
2	Feeling not to be in expert’s hands when being treated by a physician/health professional	Patients searched for the best and most trustworthy physician/health professional. They had less trust in physicians/health professionals when: physicians appeared to be young regarding their age; when physicians disagreed with the opinions of other physicians; or when a physician consulted another physician for advice.	*At the outpatient clinic, two doctors said different things—then I was confused what I should do. Then, I decided not to come to the next appointment anymore* (participant no. 28, female, age 43, Graz). *The young, unexperienced doctors always want to prescribe drugs* [DMARDs]*, but if that does not work then they are immediately at a loss, do not know what to do and then I simply do not feel well* (participant no. 165, male, age 70, Vienna).	Motivation
3	Excessive self-control	Patients who perceived excessive self-control over the treatment were less adherent.	*When the symptoms are more severe I go to see the doctor, but if they are only mild then I treat them by myself, because I know what will help anyway* (participant no. 182, female, age 34, Vienna). *It has been a long time since I was at the outpatient clinic. The drug made me uncomfortable. I vomited a lot. I never stopped taking it, because I need it. But I reduced it by myself to half the amount that the doctor had prescribed. The reduction did not affect the pain and I stopped feeling uncomfortable* (participant no. 170, female, age 45, Vienna).	Opportunity, with a negative connotation (not using the opportunity)
4	Missing a holistic approach	Some patients did not feel properly taken care of if physicians only prescribed medicines without addressing non-pharmacological aspects of treatment, including life-style advice, physical activity and diet, as well as alternative therapies.	*All I got at the outpatients clinic was medication. Nothing else. I did water gymnastics with my daughter—that was very beneficial for me, as well as mud treatments* (participant no. 99, female, age 56, Vienna). *There are also recommendations, for example regarding diet. That is never mentioned. Also regarding sports. The patients have to find out these things for themselves. They are only instructed us regarding medication here* (participant no. 182, female, age 34, Vienna).	Motivation

The capability, opportunity, and motivation model of behaviour (COM-B) model [21] was used as a frame of reference

DMARD disease-modifying anti-rheumatic drug

### Differences between medicine and non-pharmacological non-adherence

Most concepts were related to both non-adherence to medicines and non-adherence to non-pharmacological methods. However, their degree of influence was different. For example, the impact of the concept “non-adherence due to too much effort and time required” was described by patients who reported long waiting times at the rheumatology clinic. However, even greater efforts were described to implement non-pharmacological methods in daily life. Wearing splints, performing exercises, modifying life-style and/or changing daily habits were experienced to be more time consuming, were perceived as needing substantial changes of habits, and were reported to be more difficult to be included in daily patterns than taking medications. As an example, participant no. 168 (female, age 69, Vienna) explained:

*“I was told to do full-body exercises [*in German: Ganzkörperübungen*]. I do these according to my own decision. (…) There are so many things going on in my life. When I have little time, I consider them* [the exercises] *not so important. And then I just don’t do them.”*

Strong opinions of patients were primarily found with regards to medications. Patients stopped following treatment instructions because it was not in line with their preferences, values, and beliefs, e.g. pain or inflammation were seen as natural processes, and physicians were considered to be influenced by industry. Environmental factors outside the control of the patients were mentioned regardless of medicines or non-pharmacological interventions. As an example, participant no. 99 (female, age 55, Vienna) argued:

*“I do not drive and I have to wait until he* [my husband] *is well again to bring me to the clinic. (…) Initially, I wanted to take the ambulance, but it costs a lot of money and I cannot afford that.”*

### Time perspective in relation to treatment phase

Some factors associated with non-adherence were more frequently perceived by the patients in the earlier phases of the disease (all included patients had at least four visits and one DMARD prescription), while others were found more relevant in later phases of the disease. For example, not understanding the purpose or not experiencing a potential benefit were associated with non-adherence especially in the early stages of RA, as patients perceived a potential worsening of the disease, adverse events of medications, and/or no benefits of the treatment. The concept regarding excessive self-control was found in the later phases of treatment only.

### Time perspective in relation to age

A time perspective emerged in relation to age. Some patients considered themselves too old to do exercises (participant no. 110, female, age 70, Vienna; Table 2), while other patients argued that they were too young to take medication. An example is participant no. 115 (female, age 50, Vienna):

*“It was the hopelessness, a bit, that drove me away. When you are in your mid-thirties and they* [the rheumatologists] *tell you that you have to take strong medication all your life. There must be another way; I do not want to poison my body for such a long time.”*

## Discussion

We identified new and unexpected insights on factors associated with non-adherence regarding pharmacological and non-pharmacological methods in patients with RA by means of qualitative research. Moreover, we systematically described reasons for non-adherence to clinical follow-up visits, and linked them to the COM-B model. Qualitative research is a means to elicit meanings of concepts to individual patients and thus to explore reasons and motivation for behaviour [47]. We therefore decided to first start with a qualitative analysis rather than setting out to explore the influence of clinical variables on non-adherence in a statistical model. Furthermore, each individual perspective adds to the range of experiences collected. While qualitative research does not produce representative results for all patients, it allows us to better understand potential reasons for the behaviour of non-adherent patients and gives us tools to explore these in other patients. The COM-B model comprehensively covered the range of our findings. Other theories and models, such as the necessity-concerns framework [24, 25], include only parts of the concepts that emerged from our study, e.g. patients’ lack of understanding regarding the purpose of a medication or non-pharmacological method. In contrast, environmental factors, e.g. lack of supportive environmental factors, were not covered. The concept of lack of supportive environmental factors was linked to opportunity of the COM-B model. While the needs and concerns of patients have an important influence on increasing or decreasing adherence rates, other factors, such as the environment, also impact on adherence. The WHO stated that the common belief that patients themselves are solely responsible for adherence is misleading and excludes other potentially influencing factors [9]. In this sense, our study provides additional evidence to support this WHO statement.

Some findings that emerged in our qualitative analysis have already been mentioned in the literature. We already know that patient beliefs [25, 30, 33, 34, 59], patient trust [33, 34], and self-control [60], for example, influence adherence. However, we could describe these findings in greater depth regarding the perspective of patients and their motivations not to adhere to follow-up visits and recommended treatment. While beliefs, expectations, and perceptions about medication and illness have been reported in some studies [30, 33, 34, 59], quality and theoretical depth of these concepts, such as the role of pain, inflammation, or of the pharmaceutical industry from the perspective of patients, were added in our study. Therefore, the factors found in our study can contribute substantially to the ongoing debate on how and what to assess regarding (non-)adherence from the perspective of patients.

Personalized medicine claims that we need stratified interventions relevant to subgroups of patients based on biomarkers. In addition to biomarkers, psycho-social markers, including personal attitudes, strong opinions, cultural values and norms, environmental factors, and so forth, derived from qualitative data such as from our study, could be used to further stratify patients. The idea of stratifying patients for tailored patient information and education is an obvious consequence from our findings. However, a large body of research on interventions to increase adherence found that most of these interventions were not successful, although some of them have already used tailored information and targeted interventions [61]. According to the findings of our study, interventions could address different components. Interventions could target the way physicians and/or health professionals interact with patients, and care processes could be standardised to avoid disagreement and to guide younger, less experienced personnel. Furthermore, the range of patient experiences might be used as examples that could be explicitly addressed in the interactions with patients. Moreover, a complex phenomenon, such as adherence, might require multi-component interventions to successfully change human behaviour [9]. Multi-component interventions were found to be more effective in clinical trials than interventions that focused on single components only [54].

The time perspective detected in our analysis relates to two different aspects. First, patients reported differences between concepts that are important in early versus late phases of treatment. Second, a relationship with age occurred. From this we could conclude that non-adherence might not be stable throughout a patient’s lifetime, but might change in relation to experiences, values, beliefs, and the needs of patients over time. Interventions specifically targeted to the values, beliefs, and needs of a patient in certain phases of their disease course may thus be essential to sustainably influence adherence over a lifetime.

A limitation of our study is that the results are based on the perspectives of patients who were non-adherent to follow-up visits. We are aware that this is a specific subpopulation of non-adherent patients. It is very likely that some people regularly come to the outpatient clinic and are still non-adherent to their medication. These people might have other drivers for their non-adherence compared with the patients who do not show up at the outpatient clinic regularly. However, we needed this approach to identify a substantial number of patients potentially non-adherent to medicines and non-pharmacological treatment as simply asking patients would lead to socially desirable answers. In addition, future studies could explore the perspectives and motivations of those patients who have been adherent for several years. Our findings could then be compared with the perspectives of these adherent patients.

## Conclusions

In conclusion, new insights on factors associated with non-adherence allow a better understanding of this phenomenon and can substantially enhance patient care by helping to develop targeted interventions. Clinicians could explicitly address the issues during a consultation or in a patient education session. Furthermore, these new insights on factors can contribute substantially to the ongoing debate on how and what to assess regarding non-adherence from the perspective of patients.

## Additional file

Additional file 1: **Supplement S1.** Example of the qualitative analysis. **Supplement S2.** Interview guidelines used in this study. **Supplement S3.** Baseline characteristics of patients who agreed to participate in the qualitative interviews and the non-participating individuals. **Supplement S4.** The consolidated criteria for reporting qualitative research (COREQ) checklist [62]. (DOCX 80 kb)
