# Supplementary material for: I do not want to suppress the natural process of inflammation: new insights on factors associated with non-adherence in rheumatoid arthritis
Source: Arthritis Res Ther. 2018 Oct 19;20:234. doi: 10.1186/s13075-018-1732-7 (PMC6235214; doi:10.1186/s13075-018-1732-7)
Supplement: Supplementary file 1 — Supplement S1. Example of the qualitative analysis. Supplement S2. Interview guidelines used in this study. Supplement S3. Baseline characteristics of patients who agreed to participate in the qualitative interviews and the non-participating individuals. Supplement S4. The consolidated criteria for reporting qualitative research (COREQ) checklist [62]. (DOCX 80 kb) [file 13075_2018_1732_MOESM1_ESM.docx]

**Additional file 1**

*Supplement S1: The COnsolidated criteria for REporting Qualitative research checklist*

**Table A. COREQ (COnsolidated criteria for REporting Qualitative research) Checklist [**[**1**](#_ENREF_1)**] to enable the reader to quickly check the quality of this paper.**

| **Topic** | **Item no.** | **Guide Questions/’Description** | **Reported in section** |
| --- | --- | --- | --- |
| **Domain 1: Research team and reflexivity** | | | |
| **Personal characteristics** | | | |
| Interviewer/facilitator | 1 | Which author/s conducted the interview or focus group? | The first two authors conducted all interviews. This is reported in the methods section:  *To further reduce such reporting bias during the qualitative interviews, all interviews were performed by health professionals (VR, male, MMSc, health scientist with background in occupational health and therapy, as well as assistive technologies; AL, female, PhD, MSc, nursing scientist) who were not involved in the patients’ care nor otherwise related to the patients.* |
| Credentials | 2 | What were the researcher’s credentials? E.g. PhD, MD |  |
| Occupation | 3 | What was their occupation at the time of the study? |  |
| Gender | 4 | Was the researcher male or female? |  |
| Experience and training | 5 | What experience or training did the researcher have? | The interviews and the data-analysis was primarily done by VR and AL, reviewed by a patient research partner and three colleagues with extensive experience in the field of qualitative research. This is reported in the methods section:  *25% of the results were reviewed by a second researcher (either TS, EM or MO) who had extensive experience in the field of qualitative research prior to the present project [*[*2-4*](#_ENREF_2)*]. In addition, a patient research partner (ML) reviewed the results.* |
| **Relationship with participants** | | | |
| Relationship established | 6 | Was a relationship established prior to study commencement? | There was no relationship between the interviews and the participants prior to the study. This is reported in the methods section:  *To further reduce such reporting bias during the qualitative interviews, all interviews were performed by health professionals (VR, male, MMSc, health scientist with background in occupational health and therapy, as well as assistive technologies; AL, female, PhD, MSc, nursing scientist) who were not involved in the patients’ care nor otherwise related to the patients.* |
| Participant knowledge of the interviewer | 7 | What did the participants know about the researcher? e.g. personal goals, reasons for doing the research | The participants were informed comprehensively about the goals and the content of the study. They had to give oral and written consent to be included in this study. This is reported in the methods section, but also mentioned in the declaration section:  *After comparing our data to the clinical case records and the death data registry to eliminate potentially other reasons for non-adherence, such as significant other diseases or death, we contacted all identified, remaining patients via telephone, informed them about the purpose and procedures of the study, documented self-reported reasons for non-adherence to the follow up visits and invited them to participate in a qualitative semi-structured interview (conducted from April 2015 to February 2016).* |
| Interviewer characteristics | 8 | What characteristics were reported about the interviewer/facilitator? e.g. Bias, assumptions, reasons and interests in the research topic |  |
| **Domain 2: Study design** | | | |
| \| **Theoretical framework** \| \| --- \| | | | |
| Methodological orientation and theory | 9 | What methodological orientation was stated to underpin the study? e.g. grounded theory, discourse analysis, ethnography, phenomenology, content analysis | We used a meaning condensation analysis. This is reported in the methods section:  *Qualitative data were analysed by using a meaning condensation analysis [*[*3*](#_ENREF_3)*]*. |
| **Participant selection** | | | |
| Sampling | 10 | How were participants selected? e.g. purposive, convenience, consecutive, snowball | We included all patients who agreed to participate in this study. This is reported in the methods section:  *A study was performed including two parts. Firstly, we identified potentially non-adherent patients and extracted their clinical data retrospectively by using a database query at two rheumatology centres in Austria. Secondly, we invited these patients to participate in a qualitative interview.* |
| Method of approach | 11 | How were participants approached? e.g. face-to-face, telephone, mail, email | All patients were approached via telephone. This is reported in the methods section:  *After comparing our data to the clinical case records and the death data registry to eliminate potentially other reasons for non-adherence, such as significant other diseases or death, we contacted all identified, remaining patients via telephone, informed them about the purpose and procedures of the study, documented self-reported reasons for non-adherence to the follow up visits and invited them to participate in a qualitative semi-structured interview (conducted from April 2015 to February 2016).* |
| Sample size | 12 | How many participants were in the study? | Information about the sample and sample size is provide in the results section and in Fig. 1:  *Of the 459 identified patients, 32 (7 %) had died; 4 (0.9%) had a predominant other disease, including malignancies (1; 0.2%), dementia (2; 0.4%) or were in palliative care (1; 0.2%); 134 patients (29.2%) could not be reached; 27 patients (5.9%) rejected participation in the qualitative interview and 131 (28.5%) did not fulfil the inclusion criteria due to documentation errors despite the fact that they had been identified in the database query (Fig. 1). A total of 131 (28.5%) patients agreed to participate in the qualitative study.* |
| Non-participation | 13 | How many people refused to participate or dropped out? Reasons? |  |
| **Setting** | | | |
| Setting of data collection | 14 | Where was the data collected? e.g. home, clinic, workplace | Interviews were conducted via telephone or as face-to-face interviews at the out-patient’s clinic, according to the preferences of the participants. All interviews were conducted one-on-one. The following paragraph is reported in the methods section:  *If a patient gave oral and written consent to participate in this study, an appointment for a one-time, one-on-one interview was made according to the preferences of the patient either in the course of a (second) scheduled telephone call or a face-to-face interview at the clinic.* |
| Presence of non-participants | 15 | Was anyone else present besides the participants and researchers? |  |
| Description of sample | 16 | What are the important characteristics of the sample? e.g. demographic data, date | The baseline characteristics of the sample are reported in Table 1. |
| **Data collection** | | | |
| Interview guide | 17 | Were questions, prompts, guides provided by the authors? Was it pilot tested? | The whole interview guide is provided in Additional file 1 and examples of interview questions are displayed in the methods section. We had one patient research partner who reviewed and adapted the interview guide, reported in the methods section:  *An interview guide was developed for the semi-structured, individual interviews using the capability, opportunity and motivation of the COM-B model [*[*5*](#_ENREF_5)*] as a frame of reference. The interview guide was reviewed and adapted by the patient research partner (ML).* |
| Repeat interviews | 18 | Were repeat interviews carried out? If yes, how many? | The interviews were not repeated, as the time point of interest has already occurred (being non-adherent) and we thought that there will be no further benefit repeating the interviews. This is reported in the methods section:  *If a patient gave oral and written consent to participate in this study, an appointment for a one-time, one-on-one interview was made according to the preferences of the patient either in the course of a (second) scheduled telephone call or a face-to-face interview at the clinic.* |
| Audio/visual recording | 19 | Did the research use audio or visual recording to collect the data? | Interviews were all audiotaped and transcribed verbatim. If participants provided information during the first telephone contact, field notes were taken. This is reported in the methods section:  *Firstly, the audiotaped interviews were transcribed. If participants provided information during the first telephone contact, field notes were taken. The transcribed interviews and potential field notes taken during the interviews were read through to gain an overview of the collected data.* |
| Field notes | 20 | Were field notes made during and/or after the interview or focus group? |  |
| Duration | 21 | What was the duration of the interviews or focus group? | This is reported in the result section:  *Interview duration ranged from a few sentences up to a maximum of 32 minutes.* |
| Data saturation | 22 | Was data saturation discussed? | All eligible patients who agreed to participate in this study were included (this is reported in the methods section). |
| Transcripts returned | 23 | Were transcripts returned to participants for comment and/or correction? | According to our opinion and previous negative experiences (lay study participants practised to speak in whole sentences after having reviewed their transcripts), we decided on purpose not to return the transcripts to the participants for comments and/or corrections. However, a patient researcher partner (ML) reviewed data analysis to ensure that the results covered patients’ views (reported in the methods section):  *In addition, a patient research partner (ML) reviewed the results*. |
| **Domain 3: analysis and findings** | | | |
| **Data analysis** | | | |
| Number of data coders | 24 | How many data coders coded the data? | The data coding and analysis was primarily done by VR. Three other researchers and one patient research partner reviewed 25% of the data (in the methods section):  *25% of the results were reviewed by a second researcher (either TS, EM or MO) who had extensive experience in the field of qualitative research prior to the present project [*[*2-4*](#_ENREF_2)*]. In addition, a patient research partner (ML) reviewed the results.* |
| Description of the coding tree | 25 | Did authors provide a description of the coding tree? | An example of a coding tree is provided in Additional file 1:  *The following section describes an example of the qualitative analysis. “I have to take the medicines regularly, otherwise I have so much pain.”; “I also received a splint from the occupational therapist. But it did not help me and therefore, I did not use it.” and “The night splint is a painkiller – that’s why I use it regularly. It feels comfortable.” were so-called meaning units. All themes contained in the meaning units were extracted (taking medicine regularly because of pain; using/not-using a splint regularly only in case of a potential benefit for the patient, e.g. a reduction of pain) and grouped to form concepts which represent the factors associated with non-adherence, e.g. “Lack of understanding the purpose; no benefit and/or adverse events”.* |
| Derivation of themes | 26 | Were themes identified in advance or derived from the data? | The identified themes were derived from the data. This inductive data analysis is reported in the methods section:  *Qualitative data were analysed by using a meaning condensation analysis [*[*3*](#_ENREF_3)*]. Firstly, the audiotaped interviews were transcribed. If participants provided information during the first telephone contact, field notes were taken. The transcripts and potential field notes taken during the interviews were read through to gain an overview of the collected data. Secondly, the data was divided into meaning units (defined as specific units of text, a few words or a few sentences with a common meaning). Meaning units represented the range of patient experiences. In a third final step, the concepts contained in the meaning units were identified. An example to illustrate the procedure of the qualitative analysis is shown in Additional file 1: Supplement S1. The concepts depict the factors associated with non-adherence identified in our study.* |
| Software | 27 | What software, if applicable, was used to manage the data? | We used Microsoft Office to transcribe the interviews and work with the data. We conducted the descriptive analyses in SPSS 24 (reported in the methods section). We did not use any additional software to analyse the qualitative data. |
| Participant checking | 28 | Did participants provide feedback on the findings? | A patient research partner provided feedback on the findings (reported in the methods section):  *In addition, a patient research partner (ML) reviewed the results.* |
| **Reporting** | | | |
| Quotations presented | 29 | Were participant quotations presented to illustrate the themes/findings? Was each quotation identified? e.g. participant number | Quotations are presented in the results section and in Tables 2 and 3. Each quotation was indexed using participant number, gender, age and institution. For example:  *...* I have no pain anymore *(Participant No. 48, female, age 56, Graz).* |
| Data and findings consistent | 30 | Was there consistency between the data presented and the findings? | We endeavoured to ensure consistency between the data presented and the findings by using quotes to support our interpretations/findings. Please see the results section, Tables 2 and 3, for example:  *… (iv) if patients were not actively involved in a shared decision-making process. A non-adherent patient described that a shared decision about her medication treatment had not taken place:*  “If the doctor does not listen to me or does not take my opinion into account when deciding about the medication that I should take, then I change the amount of the medication myself and I potentially lie to him.” *participant No. 182 (female, aged 34, Vienna)* |
| Clarity of major themes | 31 | Were major themes clearly presented in the findings? | We presented our themes in Table 2 and 3 using a description and quotations for every theme and linked them to the COM-B model. Diverse cases, e.g. a range of different opinions, views and preferences of the participants are represented in the quotes. Differences were found between different age groups and reported in the results section in the manuscript. Minor concepts are addressed in the discussion and refer to the range of patients’ experience:  *Furthermore, the range of patient experiences might be used as examples that could be explicitly addressed in the interactions with patients.* |
| Clarity of minor themes | 32 | Is there a description of diverse cases or discussion of minor themes? |  |

*Supplement S2: Interview guideline used in this study*

***Table B. Interview guideline used in this study.*** *This table summarizes the interview questions and the theoretical aspects of the COM-B they refer to.*

| **Interview question** | ***COM-B-Component: This/these question(s) …*** |
| --- | --- |
| When you think of your last visit to the outpatient's clinic - please describe your experience from your last visit at the rheumatology centre? | Addresses issues in relation to *opportunity* (e.g. communication/relationship with health professional/physician), and *motivation* (e.g. mood state/disorder, beliefs about treatment), depending on the preferences of the patient. |
| Which medications were prescribed at your last visit in our centre and which of these do you still take?  (Probing question) What experiences have you had with these prescriptions? | Focus on the components *opportunity* (e.g. physical characteristics of medicine, regime complexity) and *motivation* (e.g. outcome expectancies). |
| Did you implement any of these recommendations in your daily life?  (Probing question) What did you not implement? Why?  (Probing question) Can you please name reasons why you did not implement the treatment recommendations you just mentioned?  Which reasons prevented you from regular follow up visits at the rheumatology centre? | Allow the patient referring their answer to either *capability, opportunity* and/or *motivation*. |
| How active is your disease at the moment?  (Probing question) How much pain do you have at the moment?  (Probing question) Do you have the feeling that you are limited by your illness in your everyday routines? Please give examples of everyday life restrictions. | Address issues particularly in the component *motivation* – it relates to how the patient experiences the disease. |
| Have you experienced any side effects due to your medication? | Focuses on the component *motivation* – it refers to concerns about current of future adverse events. |
| What's your general opinion of medication/classical medicine? | Refers to *capability* – the comprehension of disease and treatment. |
| What was your experience with non-pharmacological prescriptions/instructions? | Focuses on topics regarding *capability* (e.g. adapt to lifestyle changes) and *motivation* (e.g. outcome expectancies). |
| In your opinion - how should treatment recommendations be designed to be implemented easily in your everyday life? | Allows the patient referring their answer to either *capability, opportunity* and/or *motivation.* |
| Can you remember - How were you been informed/educated in regards of medication or other treatment recommendations?  (Probing question) How did you experience this consultation?  Do you have any people in your life, who support you to adhere with treatment recommendations?  (Probing question) If so, who is supporting you?  (Probing question) Where do you need support? | Address the component *opportunity* – issues regarding relationship and communication between the patient and the physician/health professional, and social support. |
| What is your family's/friends' attitude towards your illness/medications/classical medicine?  (Probing question) In your opinion – how does this influence your own attitude towards treatment recommendations? | Focus on the influence from the component *opportunity* on *motivation*. Social environment and cultural beliefs (opportunity) may have an impact on motivation (perception of illness and beliefs about treatment). |
| How would an ideal visit to our outpatient’s clinic look like?  (Probing question) If you could improve something at the outpatient’s clinic, what would you change?  (Probing question) What could the outpatient’s clinic have done to support you coming in for a follow up visit? | Focus mainly on the component *opportunity* – we wanted to know how a supportive environment should be designed according to the patient’s perspective. |

*Supplement S3: Example of the qualitative analysis*

The following section describes an example of the qualitative analysis. “I have to take the medicines regularly, otherwise I have so much pain.”; “I also received a splint from the occupational therapist. But it did not help me and therefore, I did not use it.” and “The night splint is a painkiller – that’s why I use it regularly. It feels comfortable.” were so-called meaning units. All themes contained in the meaning units were extracted (taking medicine regularly because of pain; using/not-using a splint regularly only in case of a potential benefit for the patient, e.g. a reduction of pain) and grouped to form concepts which represent the factors associated with non-adherence, e.g. “Lack of understanding the purpose; no benefit and/or adverse events”.

*Supplement S4: Baseline characteristics of patients who agreed to participate in the qualitative interviews and the non-participating individuals*

***Table C. Baseline characteristics of patients who agreed to participate in the qualitative interviews and the non-participating individuals.*** *Data was extracted from the last clinical visit of each patient. Metric variables are shown in terms of mean and standard deviation. For nominal variables absolute and relative frequencies were calculated. The p-value was calculated using Chi-Square test for nominal variables, and the Mann-Whitney-U-Test for ordinal and metric variables.*

| **Clinical variables** | **Participating patients** | **Non-participating patients** | **p-value*** |
| --- | --- | --- | --- |
| Number of patients^1^, N (%) | 131 (44.9%) | 161 (55.1%) | - |
| Female, N (%) | 109 (83.2122%) | 122 (75.8%) | 0.120 |
| Age in years, mean (±SD) | 62.2 (±13.5) | 60.3 (±14.5) | 0.312 |
| Disease duration in years^2^, mean (±SD) | 11.7 (±8.6) | 10.7 (±9.0) | 0.365 |
| Treatment duration in years^3^, mean (±SD) | 9.1 (±7.2) | 10.1 (±9.1) | 0.587 |
| Health Assessment Questionnaire (HAQ), mean (±SD) | 0.8 (±0.8) | 0.9 (±0.9) | 0.181 |
| Simplified Disease Activity Index (SDAI), mean (±SD) | 8.0 (±7.4) | 12.1 (±11.6) | 0.011 |
| Clinical Disease Activity Index (CDAI), mean (±SD) | 7.9 (±7.8) | 11.0 (±10.7) | 0.024 |
| PGA, Visual Analogue Scale^4^, mean (±SD) | 28.5 (±25.9) | 34.4 (±26.6) | 0.047 |
| EGA, Visual Analogue Scale^5^, mean (±SD) | 13.2 (±15.8) | 12.9 (±14.7) | 0.984 |
| Pain, Visual Analogue Scale^6^, mean (±SD) | 29.3 (±27.1) | 30.4 (±25.6) | 0.646 |
| Swollen Joint Count (SJC32)^7^, mean (±SD) | 1.9 (±3.2) | 2.1 (±3.2) | 0.485 |
| Tender Joint Count (TJC32)^8^, mean (±SD) | 3.5 (±5.7) | 5.5 (±8.4) | 0.285 |
| Rheumatoid factor (RF+)^9^, N (%) | 66 (50.4%) | 98 (60.9%) | 0.123 |

*^1^Total N=292 patients; patients who had less than 4 visits, no DMARDs, died or other predominant diseases (N=167) were not included in these calculations.*

*^2^Disease duration refers to the time duration between the first symptoms reported by the patient and the last visit at the centres.*

*^3^Treatment duration refers to the time duration between the first and the last visit when the patients presented themselves at the centres.*

*^4^Patient global assessment of disease activity: patient self-report measure using a 100mm visual analogue scale [*[*6*](#_ENREF_6)*]*

*^5^Evaluator global assessment of disease activity: in addition to the PGA, integrates subjective and objective measures, obtained by the evaluator [*[*6*](#_ENREF_6)*]*

*^6^Pain using a 100mm visual analogue scale*

*^7^Swollen Joint Count, using a 32 joint count*

*^8^Tender Joint Count, using a 32 joint count*

*^9^Rheumatoid factor positive*

**Bonferroni corrected; significant at < 0.0038*

References

1. Tong, A., P. Sainsbury, and J. Craig, *Consolidated criteria for reporting qualitative research (COREQ): a 32-item checklist for interviews and focus groups.* International journal for quality in health care, 2007. **19**(6): p. 349-357.

2. Stack, R.J., et al., *Perceptions of risk and predictive testing held by the first-degree relatives of patients with rheumatoid arthritis in England, Austria and Germany: a qualitative study.* BMJ Open, 2016. **6**(6): p. e010555.

3. Stamm, T.A., et al., *Concepts of functioning and health important to people with systemic sclerosis: a qualitative study in four European countries.* Annals of the Rheumatic Diseases, 2011. **70**(6): p. 1074-9.

4. van Beers-Tas, M.H., et al., *Initial validation and results of the Symptoms in Persons At Risk of Rheumatoid Arthritis (SPARRA) questionnaire: a EULAR project.* RMD Open, 2018. **4**(1): p. e000641.

5. Jackson, C., et al., *Applying COM-B to medication adherence: a suggested framework for research and interventions.* European Health Psychologist, 2014. **16**(1): p. 7-17.

6. Aletaha, D. and J. Smolen, *The Simplified Disease Activity Index (SDAI) and the Clinical Disease Activity Index (CDAI): a review of their usefulness and validity in rheumatoid arthritis.* Clinical and Experimental Rheumatology, 2005. **23**(5): p. 100-108.
